# Activation of the Wnt/β-Catenin Signaling Pathway by Mechanical Ventilation Is Associated with Ventilator-Induced Pulmonary Fibrosis in Healthy Lungs

**DOI:** 10.1371/journal.pone.0023914

**Published:** 2011-09-15

**Authors:** Jesús Villar, Nuria E. Cabrera, Francisco Valladares, Milena Casula, Carlos Flores, Lluís Blanch, María Elisa Quilez, Norberto Santana-Rodríguez, Robert M. Kacmarek, Arthur S. Slutsky

**Affiliations:** 1 CIBER de Enfermedades Respiratorias, Instituto de Salud Carlos III, Madrid, Spain; 2 Research Unit, Hospital Universitario Dr. Negrin, Las Palmas de Gran Canaria, Spain; 3 Keenan Research Center at the Li Ka Shing Knowledge Institute of St. Michael's Hospital, Toronto, Canada; 4 Department of Anatomy, Pathology and Histology, University of La Laguna, Tenerife, Spain; 5 Research Unit, Hospital Universitario NS de Candelaria, Santa Cruz de Tenerife, Spain; 6 Critical Care Center, Corporació Sanitaria Parc Taulí, Sabadell, Barcelona, Spain; 7 Department of Thoracic Surgery, Hospital Universitario Dr. Negrín, Las Palmas de Gran Canaria, Spain; 8 Department of Respiratory Care, Massachusetts General Hospital, Boston, Massachusetts, United States of America; 9 Department of Anesthesia, Harvard University, Boston, Massachusetts, United States of America; 10 Interdepartmental Division of Critical Care Medicine, University of Toronto, Toronto, Canada; 11 Adjunct Professor, King Saud University, Riyadh, Saudi Arabia; Northwestern University Feinberg School of Medicine, United States of America

## Abstract

**Background:**

Mechanical ventilation (MV) with high tidal volumes (V_T_) can cause or aggravate lung damage, so-called ventilator induced lung injury (VILI). The relationship between specific mechanical events in the lung and the cellular responses that result in VILI remains incomplete. Since activation of Wnt/β-catenin signaling has been suggested to be central to mechanisms of lung healing and fibrosis, we hypothesized that the Wnt/β-catenin signaling plays a role during VILI.

**Methodology/Principal Findings:**

Prospective, randomized, controlled animal study using adult, healthy, male Sprague-Dawley rats. Animals (n = 6/group) were randomized to spontaneous breathing or two strategies of MV for 4 hours: low tidal volume (V_T_) (6 mL/kg) or high V_T_ (20 mL/kg). Histological evaluation of lung tissue, measurements of WNT5A, total β-catenin, non-phospho (Ser33/37/Thr41) β-catenin, matrix metalloproteinase-7 (MMP-7), cyclin D1, vascular endothelial growth factor (VEGF), and axis inhibition protein 2 (AXIN2) protein levels by Western blot, and WNT5A, non-phospho (Ser33/37/Thr41) β-catenin, MMP-7, and AXIN2 immunohistochemical localization in the lungs were analyzed. High-V_T_ MV caused lung inflammation and perivascular edema with cellular infiltrates and collagen deposition. Protein levels of WNT5A, non-phospho (Ser33/37/Thr41) β-catenin, MMP-7, cyclin D1, VEGF, and AXIN2 in the lungs were increased in all ventilated animals although high-V_T_ MV was associated with significantly higher levels of WNT5A, non-phospho (Ser33/37/Thr41) β-catenin, MMP-7, cyclin D1, VEGF, and AXIN2 levels.

**Conclusions/Significance:**

Our findings demonstrate that the Wnt/β-catenin signaling pathway is modulated very early by MV in lungs without preexistent lung disease, suggesting that activation of this pathway could play an important role in both VILI and lung repair. Modulation of this pathway might represent a therapeutic option for prevention and/or management of VILI.

## Introduction

Although mechanical ventilation (MV) provides essential life support for critically ill patients, it can also cause or aggravate lung damage, a phenomenon called ventilator-induced lung injury (VILI). Insights into the physiology of VILI come from animal studies demonstrating that MV with larger tidal volumes (V_T_) rapidly resulted in pulmonary changes that mimic acute lung injury (ALI) and the acute respiratory distress syndrome (ARDS) [1]. The association between ALI, ventilator management, outcome, and inflammation has been strengthened in many basic and clinical studies [2]–[5]. However, despite a great deal of data suggesting interactions between mechanical stress, inflammation, and the development of lung injury, the pathogenesis of VILI is not well understood. Several physiological systems have been identified in the evolution of VILI, including coagulation, surfactant, stress response, cytoskeletal structure, oxidative injury, and endothelial/epithelial barrier dysfunction [4], [6]–[8]. Despite these advances, understanding of the relationship between specific mechanical events in the lung and the cellular responses that result in VILI remains incomplete.

Depending on the nature of the injury, lung repair mechanisms are initiated immediately following the insult. The process of lung repair is complex, involving interactions between initiating factors, structural elements, mechanical environment, and signaling pathways [9]. The role of β-catenin-mediated wingless integration (Wnt) signaling is proving to be central to mechanisms of lung healing in fibrosis [10], [11]. Wnt binding to cognate Frizzled receptors results in cytosolic accumulation of β-catenin, which then translocates to the nucleus and participates in gene transcription [9], [11]. Phosphorylation of β-catenin by glycogen synthase kinase (GSK)-3β at Ser33 and Ser37 and Thr47 targets β-catenin for ubiquitination and proteosomal degradation [12]. Accumulation of β-catenin that is specifically non-phosphorylated at these GSK3β sites is known to be critical for β-catenin-mediated transcription [13], [14]. Wnt/β-catenin signaling stimulates tissue remodeling, cell migration, and wound closure or tissue remodeling and destruction through metallopeptidases (MMPs) and other gene products. This activation directly or indirectly stimulates many of the pro-inflammatory cytokines that participate in inflammation-mediated lung destruction and thickened hyaline membranes [9]. *Mmp7* (also known as matrilysin) is a target gene of the Wnt signaling pathway found on the surface of lung epithelial cells and is known to be a key mediator of pulmonary fibrosis [15].

Since activation of Wnt/β-catenin signaling has been suggested to contribute to pulmonary fibrosis [10] in a bleomycin-induced lung injury model, we hypothesized that the Wnt/β-catenin signaling plays a role during VILI. This signaling pathway has not been tested in the context of VILI in healthy animals. To test this hypothesis we examined the activation and localization of WNT5A, β-catenin and target gene products [MMP-7, cyclin D1, vascular endothelial growth factor (VEGF), and axis inhibition protein 2 (AXIN2)] in lungs of healthy animals ventilated with low and high V_T_.

## Materials and Methods

The experimental protocol was approved by the Animal Care Committee at the Hospital Universitario Dr. Negrin, Las Palmas de Gran Canaria, Spain, in accordance with the EC Directive 86/609/EEC for animal experiments (approval ID: CEEBA-HUGCDN 003/10). A total of 18 healthy, male Sprague-Dawley rats (weight 300–325 g; CRIFFA, Barcelona, Spain) were included in the study.

### Animal preparation and experimental protocol

As previously described [5], animals were anesthetized by intraperitoneal injection of ketamine (50 mg/kg body weight) and xylazine (2 mg/kg) and randomized either to one of the two MV strategies or to no MV (n = 6 in each group). In animals allocated to MV, a cervical tracheotomy was performed using a 14G Teflon catheter. Thereafter, animals were paralyzed with 1 mg/kg pancuronium bromide and connected to a time-cycled, volume-limited rodent ventilator (Ugo Basile, Varese, Italy). Animals were ventilated for 4 hours with room air using either (i) low V_T_ (6 ml/kg) or (ii) high V_T_ (20 ml/kg) with zero positive end-expiratory pressure (PEEP). Animals were monitored non-invasively to minimize the possibility of triggering an inflammatory response by invasive procedures. In previous pilot studies using invasive monitoring, we established that this animal model resulted in adequate blood pressure and arterial blood gases.

In ventilated animals, anesthesia and paralysis were maintained by intraperitoneal injections of the ketamine/xylazine cocktail and pancuronium bromide every hour. Respiratory rate was set at 90 breaths/minute and 30 breaths/minute with low V_T_ and high V_T_, respectively. Preliminary studies showed that these respiratory settings resulted in normal PaCO_2_ values after 4 h of MV. Peak airway pressures were continuously monitored. Spontaneous breathing controls were anesthetized and observed for 4 hours. Oxygen saturation (SpO_2_) was continuously measured using a pulse oxymeter applied to the rat's tongue. During animal instrumentation and transition from spontaneous breathing to MV, SpO_2_ remained ≥90% in all animals. Rectal temperature was monitored and maintained at 36.5–37°C with radiant heat lumps. Animals were maintained supine on a restraining board inclined 20° from the horizontal. At the conclusion of the study, animals were killed by exsanguination after supplemental pentobarbital (10 mg/kg).

### Gas exchange and histological examination

At the end of the 4 h observation and ventilation period, a midline thoracotomy/laparotomy was performed. In mechanically ventilated animals, blood samples for blood gases were obtained by cardiac puncture from the beating left ventricles. The abdominal vessels were transected and the heart and lungs were removed *en bloc* from the thorax. The lungs were isolated, the trachea was cannulated, and the right lung was fixed by intratracheal instillation of 3 ml of 10% neutral buffered formalin. After fixation, the lungs were floated in 10% formalin for a week. Lungs were sampled in multiple areas, serially sliced from apex to base and specimens were embedded in paraffin, then cut (3 µm thickness), stained with hematoxylin-eosin and with the Masson-Goldner trichrome technique. The analyzing pathologist (FV) was blinded to the sample identity. Slides were viewed using a Nikon Optiphot light microscope (Tokyo, Japan) and photographed with a Nikon Digital DS-5M camera (Tokyo, Japan) at ×200 magnification. Three random sections of the right lung from each animal were examined with particular reference to alveolar and interstitial damage defined as cellular inflammatory infiltrates, pulmonary edema, atelectasis, alveolar overdistension, vascular congestion, alveolar rupture, hemorrhage and fibrosis. By scoring from 0 to 4 (none, light, moderate, severe, very severe) for each of these parameters, a total histological injury score was obtained by adding the individual scores in every animal and averaging the total values in each group [16]. A lung fibrosis score was obtained using the method of Ashcroft et al [17] and Adachi et al [18], as described previously [19]. Briefly, lung fibrosis was scored on a scale from 0 to 5 by examining the parenchyma of each sample: 0 = normal lung; 1 = minimal fibrotic thickening of alveolar or bronchial walls; 2 = moderate thickening of walls without obvious damage to the lung architecture; 3 = increased fibrosis with definite damage to the lung structure and formation of fibrous bands or small fibrous masses; 4 = severe distortion of the structure and large fibrous area; 5 = total fibrous obliteration in the field. The score was expressed as a mean score for each animal.

We used the Sirius Red staining technique [20] for quantitative morphometric assessment of collagen content, as described elsewhere [21]. With this technique, collagen fibers are stained bright red and nuclei/cytoplasms are bright yellow. Collagen fibers were measured using an image analysis program (Image J 1.42q, free software provided by the NIH, USA, and adapted for histological studies by the University of Toronto). Slides were viewed using an Olympus (BX50) microscope and were photographed with an Olympus Camedia digital camera at ×400 magnifications. Each set of data represents the percentage of collagen content compared with the total area of tissue of at least 2 fields selected randomly in different cuts.

### Western blot analysis

Left lungs were excised, washed with saline, frozen in liquid nitrogen, and stored at −80°C for subsequent protein extraction and immunoblotting. Lungs were sampled in multiple areas, homogenized, and proteins were extracted by centrifugation (14.000 rpm) for 5 min at 4°C. Protein content in the supernatant of the extract was measured with Bio-Rad DC Protein Assay. Detection of WNT5A, AXIN2 (Abcam, Cambridge, UK), total β-catenin, MMP-7, cyclin D1, and vascular endothelial growth factor (VEGF) (Santa Cruz Biotechnology, Santa Cruz, CA), non-phospho (Ser33/37/Thr41) β-catenin (Cell Signaling Technology, Massachusetts) were performed in random samples by Western blotting using rabbit polyclonal anti-WNT5A, anti-AXIN2, anti-β-catenin, anti-non-phospho (Ser33/37/Thr41) β-catenin, and anti-MMP-7 antibodies, and a goat anti-rabbit IgG-HRP as secondary antibody (Santa Cruz Biotechnology, Santa Cruz, CA), mouse monoclonal antibody anti-cyclin D1 and a rabbit anti-mouse IgG-HRP as secondary antibody (Dako, Glostrup, Denmark), goat polyclonal anti-VEFG and a donkey anti-goat IgG-HRP (Santa Cruz Biotechnology, Santa Cruz, CA). Loading control was assessed with a rabbit anti-β-actin antibody (Cell Signaling Technology, Massachusetts). In all cases, bands were detected by chemiluminescence and measured by Scion Image software package (Scion Corp., Frederick MD).

### Immnunohistochemical stainings for β-catenin, WNT5A, MMP-7 and AXIN2

Immnunohistochemical staining for WNT5A, total β-catenin, non-phospho (Ser33/37/Thr41) β-catenin, MMP-7, and AXIN2 were performed in sections from multiple lung regions according to a standard avidin-biotin-peroxidase complex protocol, as previously described [22] using a red-pink color assay (based on 3-amino-9-ethylcarbazole) that indicates positive staining for non-phospho (Ser33/37/Thr41) β-catenin. This substrate-chromogen system (AEC+ high sensitivity substrated chromogen. Dako, Glostrup, Denmark) is used in peroxidase-based immunohistochemical staining methods. A blue/violet color indicates nuclei counterstained with hematoxylin (Automation hematoxylin histological staining reagent. Dako, Glostrup, Denmark), which is suitable for visualization of nuclei in tissue and cell preparations. We used rabbit polyclonal primary antibodies against WNT5A, AXIN2 (Abcam, Cambridge, UK), anti-non-phospho (Ser33/37/Thr41) β-catenin (Cell Signaling Technology, Massachusetts), total β-catenin and MMP-7 (Santa Cruz Biotechnology, Santa Cruz, CA). Slides were viewed using an Olympus (BX50) microscope and were photographed with an Olympus Camedia digital camera at ×200, ×400 and ×1000 magnifications.

### Statistical Analyses

Values are presented as mean±SD. Comparisons involving all experimental groups were performed with one-way analysis of variance (ANOVA). We used a Bonferroni correction for multiple comparisons. Values derived from Western blot densitometry were expressed as group mean, normalized to β-actin and expressed by fold-changes of ventilated lungs vs. control (non-ventilated) lungs, and tested with the same statistical analyses. Densitometry of the non-phospho (Ser33/37/Thr41) β-catenin bands were normalized to β-actin. Densitometry of the active form (20 kDa) of MMP-7 was normalized to the inactive form (30 kDa) and then normalized to β-actin. We processed the intensity of the Sirus Red staining after conversion calculations. Data management was performed using SPSS (version 15.0 for Windows). A value of *p*<0.05 was considered significant.

## Results

### Outcome, gas exchange and pathological evaluations

All animals survived the 4-h experimental period. Mean PaO_2_ was within normal range in both groups of ventilated animals, although the mean PaO_2_ in high-V_T_ group was lower than in the low-V_T_ group (p = 0.016) (Table 1). Mean peak inspiratory pressures at the end of 4 hours of MV never exceeded 16 cm H_2_O and 28 cmH_2_O in the low-V_T_ and high-V_T_ groups, respectively.

**Table 1:** Respiratory parameters, oxygenation, histological injury scores and outcome after 4 hours of mechanical ventilation with low or high V_T_.

Parameters	Spontaneous breathing	V_T_ 6 ml/kg	V_T_ 20 ml/kg	p-value
Peak airway pressure, cmH_2_O	-	14±1	25±2	<0.0001
pH	-	7.39±0.03	7.42±0.02	0.076
PaCO_2_, mmHg	-	41.3±2	38.7±2.2	0.060
PaO_2_, mmHg	-	91±4	84±3	0.008
Histological lung injury score	0	2.1±0.3	7.8±1.1	<0.001
Lung fibrosis score	0	0.8±0.3	2.3±0.6	0.001
Mortality, %	0	0	0	-

Data are from 6 animals in each group. Numerical values are expressed as mean±SD. RR = respiratory rate; V_T_ = tidal volume.

**Figure 1:**
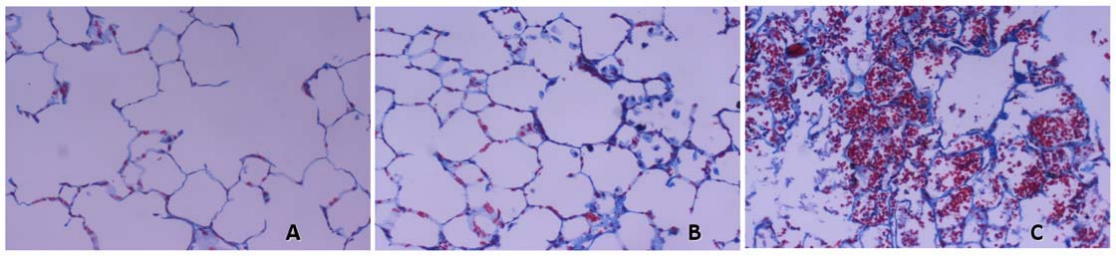
Representative histopathological features of lungs ventilated with different strategies. Panel **A**: spontaneous breathing healthy lung; panel **B**: mechanical ventilation for 4 h at low V_T_; panel **C**: mechanical ventilation for 4 h at high V_T_. Animals ventilated with high V_T_ had abundant pulmonary infiltrates, perivascular edema, alveolar hemorrhage and thickening of alveolar walls. Blue staining reveals deposition of fibrous collagen (Masson-Goldner, ×200 magnifications).

Animals ventilated with high V_T_ had greater lung damage than rats with low V_T_. After 4 h of MV with a V_T_ of 20 ml/kg, the lungs showed acute inflammatory infiltrates, perivascular edema, and collagen deposition in the parenchyma (Figure 1). The histological injury score was markedly higher in the high-V_T_ group (7.8±1.1 vs. 2.1±0.3, p<0.001) (Table 1).

**Figure 2:**
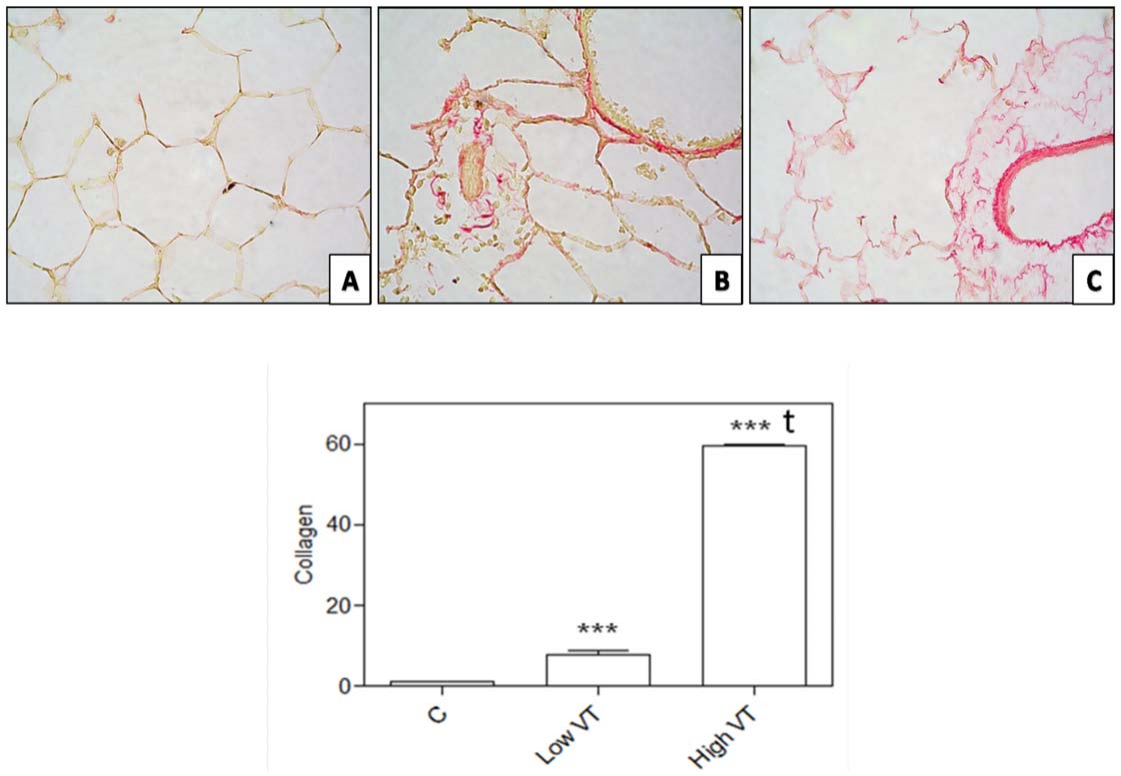
*Top:* Representative images of healthy lungs in each experimental condition. After 4 hours of spontaneous breathing (panel **A**), and after 4 hours of mechanical ventilation with low V_T_ (6 ml/kg) (panel **B**) or high V_T_ (20 ml/kg) (panel **C**) stained with the Sirius red assay for accurate collagen assessment. The staining was negative in spontaneous breathing animals. Intensity of the staining was positive in mechanically ventilated animals. Lungs ventilated with high V_T_ showed the highest collagen content. ***Down***: **Collagen content estimated from Sirius Red staining**. After conversion calculations (see text for details), animals ventilated with high V_T_ (High VT) displayed the highest intensities of collagen-rich areas in the lung compared to non-ventilated animals and animals ventilated with low V_T_ (Low VT), providing evidence for the worsening of fibrosis by high V_T_ mechanical ventilation. Images taken with ×400 magnifications. (***) p<0.001 vs. control animals; (t) p<0.001 vs. animals ventilated with low V_T_.

Independent of the MV strategy, ventilated lungs showed early signs of fibrosis at the end of 4 hours. Animals ventilated with high V_T_ had the highest fibrosis score (2.3±0.6 vs. 0.8±0.3, p = 0.001) and a marked increased of collagen deposition in the lung parenchyma (Table 1, Figure 1). Similar results were obtained with the Sirius Red staining and quantification of collagen content (Figure 2). Animals ventilated with high V_T_ displayed the highest intensities of collagen-rich areas in the lung compared to non-ventilated animals and animals ventilated with low V_T_ (p<0.001), providing evidence for the worsening of fibrosis by high V_T_ MV.

### WNT5A and β-catenin protein levels in the lungs

**Figure 3:**
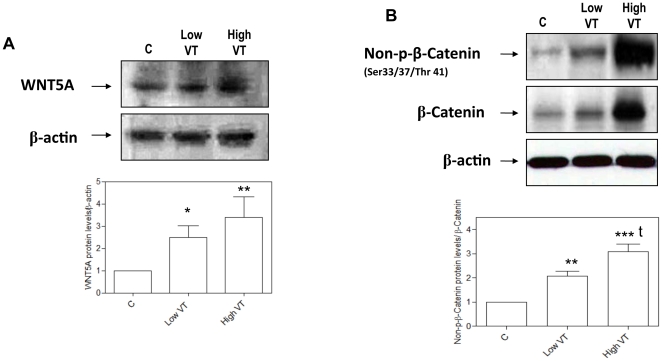
Representative gels for WNT5A and β-catenin activation. Changes in WNT5A (**A**) and β-catenin (**B**) protein levels under different ventilatory strategies: spontaneously breathing animals (control, **C**), ventilated with low V_T_ and ventilated with high V_T_. Blots were probed with WNT5A and non-phosphorylated (Ser33/37/Thr41) antibodies to β-catenin and subsequently stripped and reprobed for antibody to total β-catenin (in B) and β-actin. WNT5A densitometry bands were normalized to β-actin. Non-phosphorylated (Ser33/37/Thr41) β-catenin bands were normalized to total β-catenin and β-actin. Graphs represent data from 6 animals in each group and are expressed in relation to control, non-ventilated animals (bar C). (*) p<0.05 vs. control animals; (**) p<0.01 vs. animals ventilated with low V_T_; (***) p<0.001 vs. controls; (t) p<0.01 vs. animals ventilated with low V_T_.

WNT5A protein levels increased in ventilated animals (Figure 3A). The highest WNT5A protein levels were found in rats ventilated with high V_T_ (*p*<0.01, Figure 3A). MV resulted in increased non-phosphorylated (threonine and serine) β-catenin (Figure 3B). This increase was significantly higher in rats ventilated with high V_T_ compared to non-ventilated animals (p<0.001) and animals ventilated with low V_T_ (p<0.01). [See the full gels with molecular weight markers in Figure S1 of the Electronic Supplementary Material (ESM)].

### High V_T_ modifies immunohistochemical stainings for WNT5A and β-catenin activation

**Figure 4:**
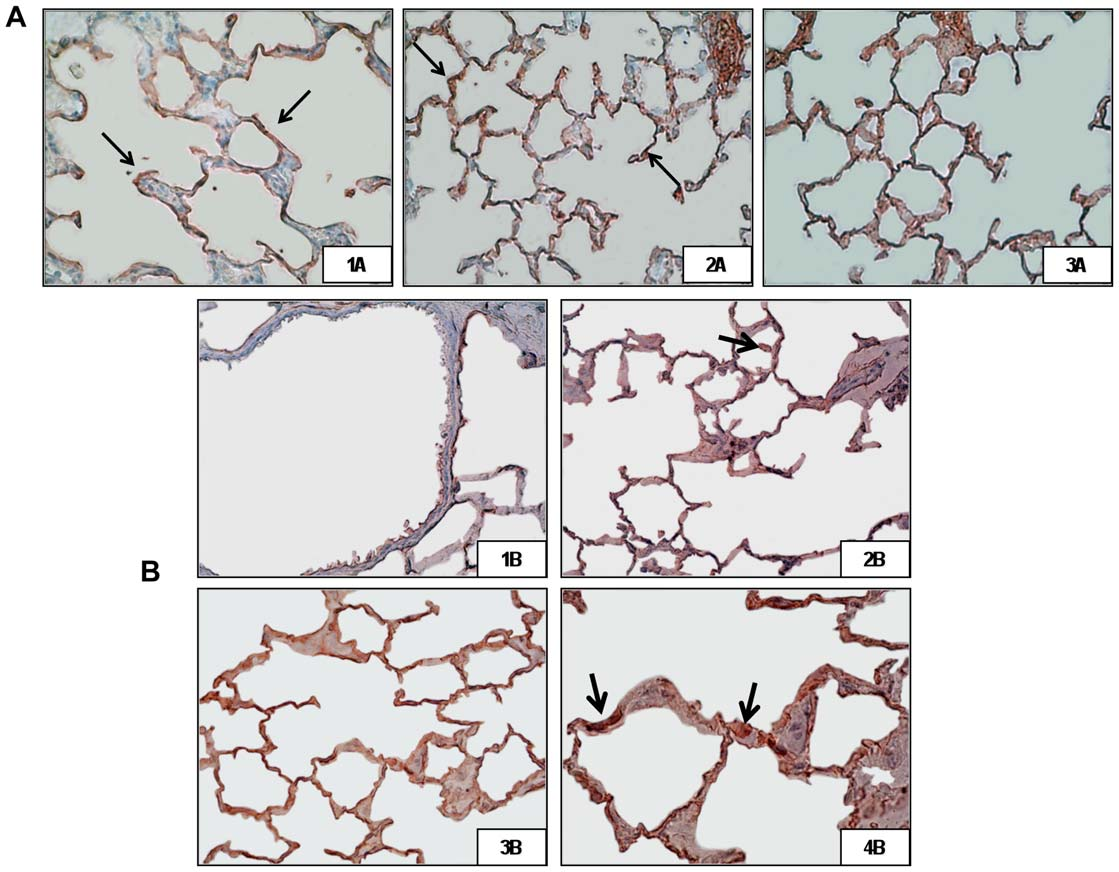
Immunohistochemical stainings for WNT5A and β-catenin activation. Immunohistochemical localization of WNT5A (**A**) and non-phospho (Ser33/37/Thr41) β-catenin (**B**) in spontaneous breathing animals (1A–B), animals ventilated with low V_T_ (2A–B) and animals ventilated with high V_T_ (3A–B, 4B). Red-pink color indicates positive staining (3-amino-9-ethylcarbazole) for WNT5A and total β-catenin proteins; blue/violet indicates nuclei counterstained with hematoxylin. WNT5A staining was found in lung septa (large arrows) and non-phospho (Ser33/37/Thr41) β-catenin staining was found in the nuclei of different cell types in lung septa (short arrows). Strong immunostaining for WNT5A was also observed in the high V_T_ rat group. Panels show a ×400 magnification and 4B panel shows a ×1000 magnification of panel 3B.

MV modified the intensity of WNT5A and β-catenin staining (Figures 4A and 4B). While in spontaneous breathing animals there was a basal intensity in alveolar walls and septa (panel 1A), this intensity was moderate in the low-V_T_ group (panel 2A). However, lungs ventilated with high V_T_ (panel 3A) showed strong and abundant WNT5A and total β-catenin staining. Non-phosphorylated β-catenin was not detected in the airway epithelium of spontaneous breathing animals (Figure 4B, panel 1B) but it was detected at the nuclei of airway epithelium in lungs ventilated with low V_T_ (panel 2B) and high V_T_ (panel 3B). This activation was maximum in animals ventilated with high VT (panel 4B).

### MMP-7, cyclin D1, VEGF, AXIN2 protein levels and MMP-7 and AXIN2 immunohistochemical stainings

**Figure 5:**
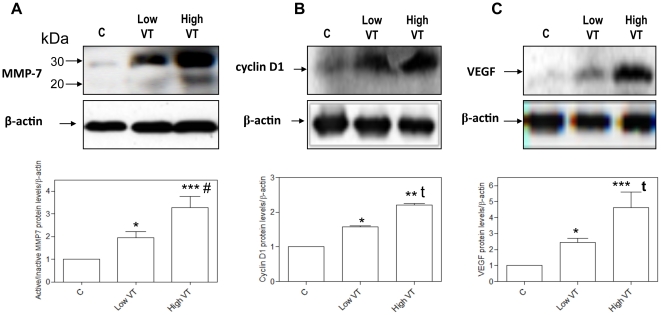
Mechanical ventilation increased MMP-7, cyclin D1, and VEGF in the lung. Densitometry analysis of the active form (20 kDa) of MMP-7 was normalized to the inactive form (30 kDa) and then normalized to β-actin. Densitometry analysis (in graph) and representative gels correspond to three different experimental protocols: spontaneous breathing animals (controls, C), ventilated with low V_T_ and ventilated with high V_T_. (*) p<0.05 vs. control animals; (**) *p*<0.01 vs. controls; (***) *p*<0.001 vs. controls; (#) p<0.05 vs. animals ventilated with V_T_; (t) *p*<0.01 *vs.* animals ventilated with low V_T_.

**Figure 6:**
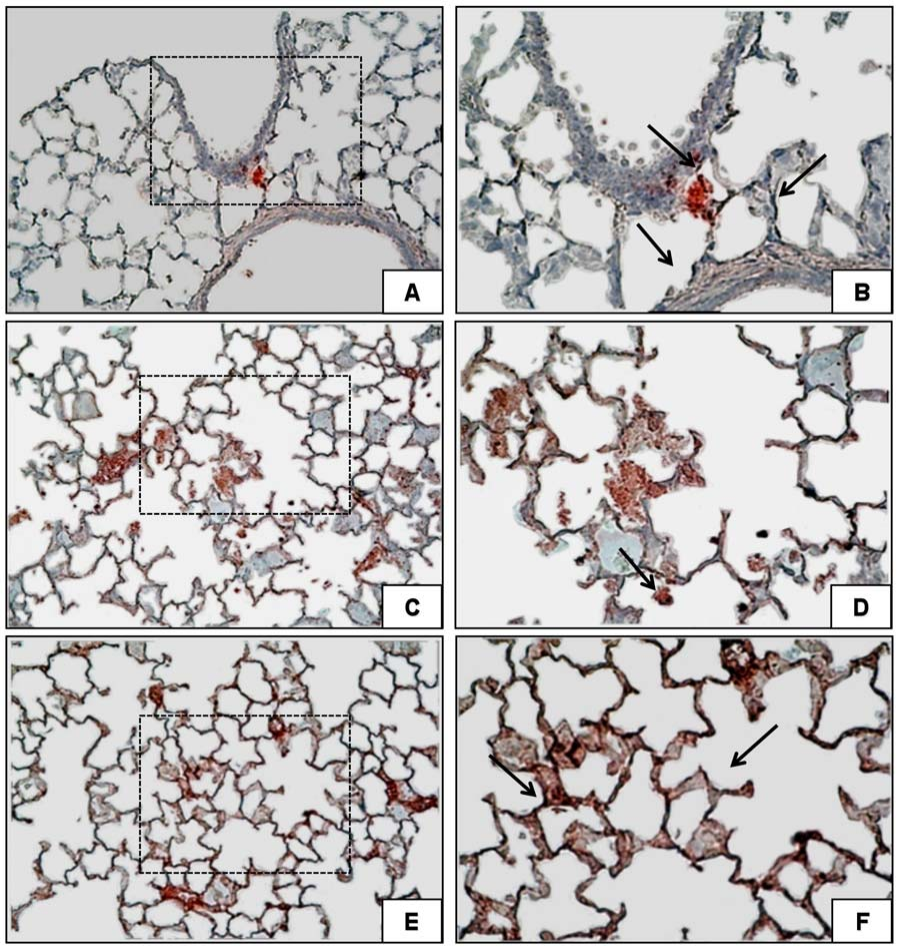
Immnohistochemical staining reveals elevated for MMP-7 expression induced by mechanical ventilation. Lung images in 5A and 5B are from spontaneous breathing rats; images in 5C and 5D are from rats ventilated with low V_T_ and in 5E and 5F are from rats ventilated with high V_T_. Red-pink color indicates positive staining for MMP-7 and blue/violet indicates nuclei counterstained with hematoxylin. MMP-7 expression was observed in alveolar walls and septa. Insets are 400× magnifications of the indicated areas (5B,5D,5F).

High-V_T_ MV caused increased upregulation of active MMP-7, cyclin D1 and VEGF (p<0.001, when compared to low V_T_ and spontaneous breathing animals) (Figure 5). We found abundant immnunostaining for MMP-7 in lung ventilated with high V_T_ (Figure 6).

**Figure 7:**
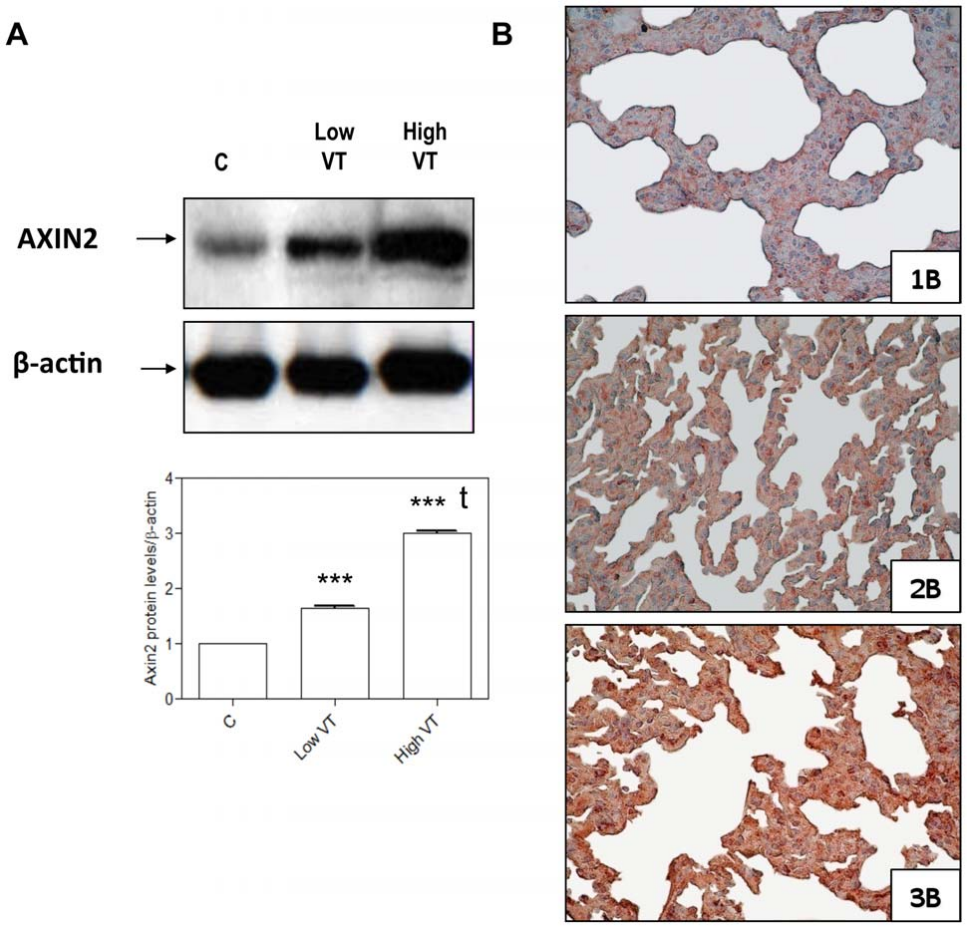
Mechanical ventilation increased AXIN2 protein levels in the lung. (**A**) Immunoblot analysis of AXIN2 expression in control, spontaneous breathing animals (C), and ventilated with low V_T_ (Low VT) or high V_T_ (High VT). The expression of β-actin was examined as a loading control. (***) p<0.001 vs. control animals; (t) p<0.001 vs. animals ventilated with low V_T_. (**B**) Representative photomicrographs of AXIN2 immunohistochemical staining in the lung of spontaneous breathing animals (1B), animals ventilated with low VT (2B), and animals ventilated with high VT (3B). The red color indicates positive staining for AXIN2 and blue/violet colors indicate nuclei counterstained with hematoxylin. ×400 magnifications.

MV induced a volume-dependent increase in AXIN2 protein levels (p<0.001, when low V_T_ vs. spontaneous breathing animals, high V_T_ vs. low V_T_, and high V_T_ vs. spontaneous breathing groups were compared) (Figure 7, panel A). ). (See the full gel with molecular weight markers in Figure S1 of the ESM). Immunohistochemistry assays showed that AXIN2 was detected at higher intensity in animals ventilated with low V_T_ compared to non-ventilated animals, and at higher intensity in animals ventilated with high V_T_ (Figure 7, panel B).

## Discussion

This study demonstrates that (i) WNT5A is expressed and localized in healthy lungs in response to MV in the absence of infection or toxics, (ii) the expression of WNT5A is dependent on the applied V_T_, (iii) activation of WNT5A and β-catenin are associated with up-regulation of downstream target genes, (such as *Mmp7*, *cyclin D1*, *Vegf*, and *Axin2*), and (iv) pulmonary fibrosis is induced very early during VILI. Although the Wnt pathway has been reported to play a role in the sustained inflammation during sepsis [23], this study is the first to provide evidence of its relevance to pathological inflammation and profibrotic transformation in VILI in otherwise normal animals. These findings support the concept that even at low V_T_, MV can induce a pro-inflammatory reaction in the absence of pre-existing lung injury [24]–[26]. Moriondo et al [24] reported that during MV of healthy animals, lung expression of inflammatory mediators were higher in lungs ventilated with high respiratory rate, suggesting that MV *per se* might represent a challenging factor for the lung parenchyma.

We were interested in examining the modulation of WNT5A, β-catenin, MMP-7, cyclin D1, VEGF, and AXIN2 because these molecules contribute to lung repair and fibrosis [9], [15], [27], [28]. β-catenin signaling can stimulate tissue remodeling, cell migration, and wound closure through MMPs or tissue destruction through MMPs and other mediators [11]. Wnt ligands induce lung epithelial cell proliferation, fibroblast activation and collagen synthesis [15]. To date, 19 different WNT proteins have been identified in humans, most of which have been shown to be expressed in a tightly regulated spatial temporal manner [29]. Several *Wnt* genes are expressed in the developing and adult lung. Of these, *Wnt5a* and *Wnt7b* are expressed at high levels exclusively in the developing and adult airway epithelium [14]. We chose to examine the modulation of WNT5A because it has been implicated in several pulmonary disorders [11], [23] and has not been studied in the context of VILI in healthy animals. In an experimental model of interstitial lung fibrosis, Vuga et al [30] showed that WNT5a was significantly increased in fibroblasts obtained from lung tissue and suggested that WNT5A may play a role in fibroblast proliferation in idiopathic pulmonary fibrosis and other fibrotic interstitial lung diseases. The activation of Wnt signaling after high-V_T_ MV likely represents a regenerative signal of the damaged epithelium [31]. Miyahara et al [32] reported that total lung β-catenin was unchanged in isolated perfused mouse lungs ventilated with low V_T_ but was up-regulated during high-V_T_ ventilation. Blumenthal et al [33] reported that the expression of WNT5A required Toll-like receptor signaling and NF-κB activation. In a previous report and using the same experimental model as in the present study, we have shown that MV *per se* is able to modulate the NF-κB activation through the Toll-like receptor signaling [22]. The cell cycle regulatory molecule cyclin D1 gene is one of the target genes for the Wnt/β-catenin signaling pathway, and VEGF is required for maintenance of adult lung alveolar structures. Our data on cyclin D1 and VEGF are in agreement with previous studies. Using epithelial cells from patients with idiopathic pulmonary fibrosis, Königshoff et al [15] showed that Western blot analysis of *Wnt* target gene products cyclin D1 and MMP-7 demonstrated increased functional Wnt/β-catenin signaling in pulmonary fibrosis compared with control patients. Similarly, Stockmann et al [27] reported that detection of VEGF protein by Western blot in whole lung lysates revealed markedly elevated VEGF levels after intraperitoneal bleomycin treatment in mice. AXIN2 (also known as conductin) is a downstream target of the Wnt/β-catenin pathway [34] and has been implicated in a bleomycin-induced ALI model [28]. All those observations and our findings support the importance of Wnt signaling in the early development of ventilator-induced fibrosis.

Our study suggests a new order of complexity in regulating and understanding the bridge between MV and VILI. Any tissue repair involves coordinated cellular infiltration together with extracellular matrix (ECM) deposition and re-epithelization. Collagen is the major structural protein of the ECM of the lung. In the first step, injured cells are replaced by cells of the same type, and then normal parenchyma is replaced by connective tissue leading to fibrosis and remodeling of the lung architecture [35]–[37]. Usually these steps are required for healing. As reported by others, MV with low V_T_ in healthy animals induces reversible pulmonary and systemic inflammatory reactions [38]. However, when the pro-inflammatory and pro-fibrotic responses become persistent or uncontrolled, the process can lead to fibrosis, organ failure and death. Proteolytic degradation of the ECM requires MMPs and gene transcription of MMPs is regulated by Wnt signaling of both canonical and non-canonical pathways. Our findings support recent observations by Moriondo et al [24] who found that healthy rats ventilated for 4 h with high V_T_ had increased pulmonary proteoglycans (a main component of the ECM) and activated MMPs. Although the proteoglycan fragmentation was V_T_-dependent, it is plausible that repetitive stretch/collapse cycles during MV at low V_T_ with elevated ventilatory rates may cause disorganization and/or remodeling of the ECM of the lung. The MMP family of proteins comprises more than 25 enzymes that cleave different components of the ECM [37]. Although the traditional role for MMPs is the cleavage of ECM substrates, such as collagen, elastin and laminin, induction of MMP activity may have numerous downstream functional consequences. Zuo et al [39] analyzed samples from patients with histologically proven pulmonary fibrosis using microarray technology and found that *Mmp7* was the most up-regulated gene, a finding that was demonstrated by immunohistochemistry. The increased MMP7 in our study supports a role of this enzyme in perpetuating lung inflammation and remodeling after MV. One might predict that interfering with the expression and production of WNT5A, its interaction with Frizzled-5 receptor, or the downstream signaling (MMP-7, cyclin D1, AXIN2) might represent a therapeutic option for limiting the development or progression of VILI in mechanically ventilated critically ill patients. In fact, *Mmp7* deficient mice are protected from intratracheal bleomycin-induced pulmonary fibrosis [39].

The present study has some limitations and strengths. First, although our data do not fully confirm that Wnt/β-catenin signaling pathway is involved in early development of fibrosis in VILI, another study has demonstrated that selective inhibition of this pathway can reverse pulmonary fibrosis [40]. Second, we did not use WNT5A deficient animals to prove that target genes of this pathway contribute to the pathogenesis of ventilator-induced fibrosis in VILI. However, other investigators [39] have reported protection from bleomycin-induced pulmonary fibrosis in *Mmp7* deficient mice. Since WNT5A is involved in the modulation of inflammatory responses induced by microbial stimulation [33], our non-infectious model (i.e. VILI) offers further support that the Wnt/β-catenin signaling pathway is involved in the process of lung repair following injury. Third, similar to the study by Moriondo et al [24], we did not apply PEEP in the experimental groups since (i) animals had healthy lungs, (ii) it has been shown that lung volume is similar during spontaneous breathing and MV at the same level of anesthesia [41], and (iii) the protective effects of PEEP have been demonstrated in animals and humans with pre-existing lung injury [42]. The aim of the present study was not to propose a new ventilatory strategy during MV but to investigate whether the application of an increased lung stress (high V_T_) would produce a response in Wnt/β-catenin signaling. However, it is possible that the minor degree of injury and collagen deposition in the lungs of low-V_T_ animals was a consequence of the lack of a moderate level of PEEP. Therefore, as Moriondo et al reported [24], MV *per se* might induce lung damage and trigger an inflammatory response due to the continuous cyclical tissue stretching, even at physiological low V_T_ values.

In conclusion, we have documented that MV for 4 hours increased protein levels of WNT5A and non-phosphorylated (threonine and serine) β-catenin in lungs of previously healthy rats. We also observed increased expression of the Wnt target gene products MMP-7, cyclin D1, VEGF, and AXIN2 that are thought to play an important role in pulmonary fibrosis. Therefore, the Wnt/β-catenin signaling pathway may represent a novel therapeutic target for prevention and management of VILI. There are still many unresolved questions regarding the role of Wnt/β-catenin in the lung repair mechanisms and a comprehensive understanding of epithelial repair in the lungs during VILI is still far from complete.

## Supporting Information

Figure S1 **Representative full gels of WNT5A, non-phosphorylated (Ser33/37/Thr41) β-catenin, and AXIN2 Western blotting with molecular weight markers.**
(TIF)

## References

[1] Dreyfuss D, Saumon G (1998). Ventilator-induced lung injury: lessons from experimental studies.. Am J Respir Crit Care Med.

[2] The Acute Respiratory Distress Syndrome Network (2000). Ventilation with lower tidal volumes as compared with traditional tidal volumes for acute lung injury and the acute respiratory distress syndrome.. N Engl J Med.

[3] Tremblay LN, Miatto D, Hamid Q, Govindarajan A, Slutsky AS (2002). Injurious ventilation induces widespread pulmonary epithelial expression of tumor necrosis factor-alpha and interleukin-6 messenger RNA.. Crit Care Med.

[4] Copland IB, Kavanagh BP, Engelberts D, McKerlie C, Belik J (2003). Early changes in lung gene expression due to high tidal volume.. Am J Respir Crit Care Med.

[5] Villar J, Herrera-Abreu MT, Valladares F, Muros M, Pérez-Méndez L (2009). Experimental ventilator-induced lung injury: exacerbation by positive end-expiratory pressure.. Anesthesiology.

[6] Abraham E (2000). Coagulation abnormalities in acute lung injury and sepsis.. Am J Respir Cell Mol Biol.

[7] Eisner MD, Parsons P, Matthay MA, Ware L, Greene K (2003). Plasma surfactant protein levels and clinical outcomes in patients with acute lung injury.. Thorax.

[8] Grigoryev DN, Ma SF, Irizarry RA, Ye SQ, Quackenbush J (2004). Orthologous gene-expression profiling in multi-species models: search for candidate genes.. Genome Biol.

[9] Crosby LM, Waters CM (2010). Epithelial repair mechanisms in the lung.. Am J Physiol Lung Cell Mol Physiol.

[10] Königshoff M, Kramer M, Balsara N, Wilhelm J, Amarie OV (2009). WNT1-inducible signaling protein-1 mediates pulmonary fibrosis in mice and is upregulated in humans with idiopathic pulmonary fibrosis.. J Clin Invest.

[11] Pongracz JE, Stockley RA (2006). Wnt signalling in lung development and diseases.. Respir Res.

[12] Cantley LC (2002). The phosphoinositide 3-kinase pathway.. Science.

[13] Staal FJ, Noort MvM, Strous GJ, Clevers HC (2002). Wnt signals are transmitted through N-terminally dephosphorylated beta-catenin.. EMBO reports.

[14] Morrisey EE (2003). Wnt signaling and pulmonary fibrosis.. Am J Pathol.

[15] Königshoff M, Balsara N, Pfaff EM, Kramer M, Chrobak I (2008). Functional Wnt signaling is increased in idiopathic pulmonary fibrosis.. PLoS One.

[16] Herrera MT, Toledo C, Valladares F, Muros M, Diaz-Flores L (2003). Positive end-expiratory pressure modulates local and systemic inflammatory responses in a sepsis-induced lung injury model.. Intensive Care Med.

[17] Ashcroft T, Simpson JM, Timbrell V (1988). Simple method of estimating severity of pulmonary fibrosis on a numerical scale.. J Clin Pathol.

[18] Adachi K, Suzuki M, Sugimoto T, Yorozu K, Takai H (2003). Effects of granulocyte colony-stimulating factor (G-CSF) on bleomycin-induced lung injury of varying severity.. Toxicol Pathol.

[19] Li LF, Liao SK, Huang CC, Hung MJ, Quinn DA (2008). Serine/threonine kinase-protein kinase B and extracellular signal-regulated kinase regulate ventilator-induced pulmonary fibrosis after bleomycin-induced acute lung injury: a prospective, controlled animal experiment.. Crit Care.

[20] Malkusch W, Rehn B, Bruch J (1995). Advantages of Sirius Red staining for quantitative morphometric collagen measurements in lungs.. Exp Lung Res.

[21] Martinez-Galan L, del Puerto-Nevado L, Perez-Rial S, Diaz-Gil JJ, Gonzalez-Mangado N (2010). [Liver growth factor improves pulmonary fibrosis secondary to cadmium administration in rats].. Arch Bronconeumol.

[22] Villar J, Cabrera NE, Casula M, Flores C, Valladares F (2010). Mechanical ventilation modulates TLR4 and IRAK-3 in a non-infectious, ventilator-induced lung injury model.. Respir Res.

[23] Pereira C, Schaer DJ, Bachli EB, Kurrer MO, Schoedon G (2008). Wnt5A/CaMKII signaling contributes to the inflammatory response of macrophages and is a target for the antiinflammatory action of activated protein C and interleukin-10.. Arterioscler Thromb Vasc Biol.

[24] Moriondo A, Pelosi P, Passi A, Viola M, Marcozzi C (2007). Proteoglycan fragmentation and respiratory mechanics in mechanically ventilated healthy rats.. J Appl Physiol.

[25] Caruso P, Meireles SI, Reis LF, Mauad T, Martins MA (2003). Low tidal volume ventilation induces proinflammatory and profibrogenic response in lungs of rats.. Intensive Care Med.

[26] Wolthuis EK, Vlaar APJ, Choi G, Roelofs JJTH, Juffermans NP (2009). Mechanical ventilation using non-injurious ventilation settings causes lung injury in the absence of pre-existing lung injury in healthy mice.. Crit Care.

[27] Stockmann C, Kerdiles Y, Nomaksteinsky M, Weidemann A, Takeda N (2010). Loss of myeloid cell-derived vascular endothelial growth factor accelerates fibrosis.. Proc Natl Acad Sci USA.

[28] Flozak AS, Lam AP, Russell S, Jain M, Peled ON (2010). Beta-catenin/T-cell factor signaling is activated during lung injury and promotes the survival and migration of alveolar epithelial cells.. J Biol Chem.

[29] Logan CY, Nusse R (2004). The Wnt signaling pathway in development and disease.. Annu Rev Cell Dev Biol.

[30] Vuga LJ, Ben-Yehudah A, Kovkarova-Naumovski E, Oriss T, Gibson KF (2009). WNT5A is a regulator of fibroblast proliferation and resistance to apoptosis.. Am J Respir Cell Mol Biol.

[31] Königshoff M, Eickelberg O (2010). WNT signaling in lung disease: a failure or a regeneration signal?. Am J Respir Cell Mol Biol.

[32] Miyahara T, Hamanaka K, Weber DS, Drake DA, Anghelescu M (2007). Phosphoinositide 3-kinase, Src, and Akt modulate acute ventilation-induced vascular permeability increases in mouse lungs.. Am J Physiol Lung Cell Mol Physiol.

[33] Blumenthal A, Ehlers S, Lauber J, Buer J, Lange C (2006). The Wingless homolog WNT5A and its receptor Frizzled-5 regulate inflammatory responses of human mononuclear cells induced by microbial stimulation.. Blood.

[34] Leung JY, Kolligs FT, Wu R, Zhai Y, Kuick R (2002). Activation of AXIN2 expression by beta-catenin-T cell factor. A feedback repressor pathway regulating Wnt signaling.. J Biol Chem.

[35] Negrini D, Tenstad O, Passi A, Wiig H (2006). Differential degradation of matrix proteoglycans and edema development in rabbit lung.. Am J Physiol Lung Cell Mol Physiol.

[36] Pelosi P, Rocco PR (2008). Effects of mechanical ventilation on the extracellular matrix.. Intensive Care Med.

[37] Cosgrove GP, du Bois RM (2008). Matrix metalloproteinase-7 expression in fibrosing lung disease: restoring the balance.. Chest.

[38] Vaneker M, Halbertsma FJ, van Egmond J, Netea MG, Dijkman HB (2007). Mechanical ventilation in healthy mice induces reversible pulmonary and systemic cytokine elevation with preserved alveolar integrity: an in vivo model using clinical relevant ventilation settings.. Anesthesiology.

[39] Zuo F, Kaminski N, Eugui E, Allard J, Yakhini Z (2002). Gene expression analysis reveals matrilysin as a key regulator of pulmonary fibrosis in mice and humans.. Proc Natl Acad Sci USA.

[40] Henderson WR, Chi EY, Ye X, Nguyen C, Tien YT (2010). Inhibition of Wnt/beta-catenin/CREB binding protein (CBP) signaling reverses pulmonary fibrosis.. Proc Natl Acad Sci USA.

[41] Tokics L, Hedenstierna G, Strandberg A, Brismar B, Lundquist H (1987). Lung collapse and gas exchange during general anesthesia: effects of spontaneous breathing, muscle paralysis, and positive end-expiratory pressure.. Anesthesiology.

[42] dos Santos CC, Slutsky AS (2004). Protective ventilation of patients with acute respiratory distress syndrome.. Crit Care.

