# Supplementary figures and images for: Activation of the Wnt/β-Catenin Signaling Pathway by Mechanical Ventilation Is Associated with Ventilator-Induced Pulmonary Fibrosis in Healthy Lungs
Source: PLoS One. 2011 Sep 15;6(9):e23914. doi: 10.1371/journal.pone.0023914 (PMC3174135; doi:10.1371/journal.pone.0023914)

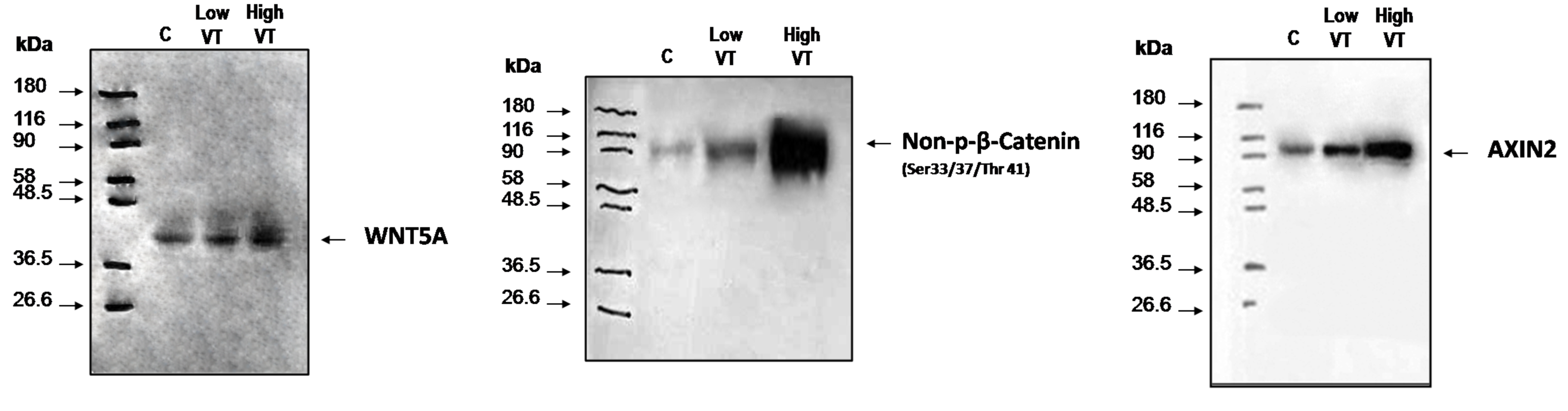

Supplement: Figure S1 — Representative full gels of WNT5A, non-phosphorylated (Ser33/37/Thr41) β-catenin, and AXIN2 Western blotting with molecular weight markers. (TIF) [file pone.0023914.s001.tif]
